# Spatial and temporal genetic heterogeneity of epidermal growth factor receptor gene status in a patient with non-small cell lung cancer: a case report

**DOI:** 10.1186/1752-1947-5-553

**Published:** 2011-11-22

**Authors:** Makoto Ogata, Toshiki Shimizu, Takashi Yokoi, Shosaku Nomura

**Affiliations:** 1 First Department of Internal Medicine, Kansai Medical University, 10-15 Fumizono-cho, Moriguchi City, Osaka 570-8506, Japan

## Abstract

**Introduction:**

To date, an epidermal growth factor receptor-activating mutation is recognized as a genetic hallmark that predicts a good response to treatment with epidermal growth factor receptor tyrosine kinase inhibitor. However, there has been less long-term observation of the mutational status within the same patient. To the best of our knowledge, this is the first case report which illustrates the instability of the genetic status of pulmonary adenocarcinoma cells.

**Case presentation:**

A 64-year-old Japanese woman with advanced lung adenocarcinoma had been undergoing various anticancer treatments, including epidermal growth factor receptor tyrosine kinase inhibitor, for seven years. She had been receiving locoregional treatment in addition to systemic treatment. She maintained a good performance status until seven years after the initial diagnosis, although she had local and distant recurrences. We analyzed the genetic status of the epidermal growth factor receptor gene in a series of specimens obtained from various tumor-containing lesions throughout the therapeutic period. The results of the genetic analyses clearly showed that the spatial and temporal genetic heterogeneity of the epidermal growth factor receptor gene status originated from an identical tumor ancestor.

**Conclusions:**

An alternative paradigm to determine a therapeutic strategy for a patient with lung cancer should be considered given the genetic heterogeneity and instability of tumor cells.

## Background

Epidermal growth factor receptor (EGFR) tyrosine kinase inhibition is an active strategy in non-small cell lung cancer (NSCLC) [1]. The response to EGFR tyrosine kinase inhibitor (EGFR-TKI) has been shown to be closely related to the somatic activating mutation of the EGFR gene in tumor cells [2]. EGFR mutation has been recognized as a crucial step in the transformation of alveolar epithelial cells. A recent report also suggested that the activating mutation of the EGFR gene occurs as an early event during carcinogenesis of lung cancer [3]. Discordance in the mutation status of the EGFR gene in the primary tumor and corresponding metastatic tumor is occasionally observed [4-6]. However, there has been less long-term observation of the mutational status of the EGFR gene in the same patient. We report a series of analyses of the EGFR gene status of a patient. The results of our analyses clearly demonstrate a spatial and temporal genetic heterogeneity, including double-activating mutation, in this patient.

## Case presentation

A 64-year-old Japanese woman was admitted to our hospital seven years ago with a complaint of pain in her right hip joint. Radiographic analysis revealed an osteolytic tumor of her right pelvis and a tumor in her right lower lung field. The histological findings of a biopsy specimen obtained from the bone and pulmonary tumors showed adenocarcinoma. Immunohistochemical tests showed that the tumor cells stained positive for thyroid transcription factor-1. Therefore, we diagnosed our patient with advanced lung cancer (cT2N2M1). She received systemic chemotherapy with carboplatin and paclitaxel, starting one month after diagnosis after palliative irradiation of the pelvic lesion. After completion of four consecutive courses of chemotherapy, a partial response was achieved. However, local recurrence occurred six months later. Because docetaxel, gemcitabine and vinorelbine were all insufficient for inhibiting disease progression, gefitinib was administered as the fourth regimen, starting one year after diagnosis. A tumor response was subsequently observed and the treatment was continued. However, a routine brain magnetic resonance imaging scan showed a *de novo *metastatic lesion in her left frontal lobe two years after diagnosis. In accordance with our patient's wishes, gefitinib administration was continued after surgical resection of the brain tumor. Although the primary lesion did not exhibit regrowth, additional brain and pulmonary metastases in her right lung were observed four years after diagnosis. Erlotinib was administered as the fifth regimen following stereotactic radiosurgery for the brain tumor. Significant growth of the pulmonary metastatic lesion was observed one year later, although the other lesions did not demonstrate regrowth. We repeated a bronchoscopy for the pulmonary metastatic lesion to investigate the EGFR gene mutation status.

**Figure 1:**
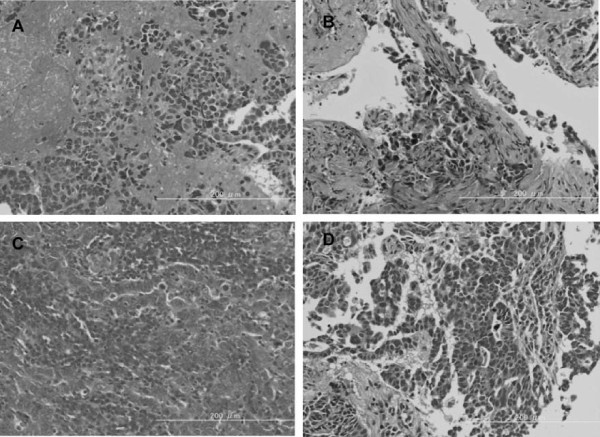
**Histological findings for the various tumor specimens, with hematoxylin and eosin staining clearly demonstrating the presence of adenocarcinoma cells**. All the original magnifications are ×200; **(A) **bone metastasis, **(B) **primary pulmonary tumor; **(C) **brain metastasis; **(D) **pulmonary metastasis.

We used a combination of the peptide nucleic acid-locked nucleic acid polymerase chain reaction (PNA-LNA PCR) clamp method and the direct sequencing method for determining the EGFR gene mutation status [7]. The result of the PNA-LNA PCR clamp assay for the EGFR gene showed a double-activating mutation consisting of an in-frame deletion mutation in exon 19 and an L858R point mutation in exon 21. The mutation identified in exon 19 was consistent with I744-R748del and two subsequent substitution mutations, E749I (GAA to ATT) and A750K (GCA to AAA). To shed light on the sequential changes in the EGFR mutation status, we also analyzed a series of paraffin-embedded samples obtained from this patient's tumors. The histological findings of the analyzed samples clearly demonstrated the presence of adenocarcinoma cells (Figure 1). The results are summarized in Table 1. Genetic analysis of the specimen from the primary pulmonary tumor at diagnosis showed a wild-type EGFR gene. The specimen from the metastatic bone tumor had an exon 19 deletion identical to that in the metastatic pulmonary tumor. L858R was not observed in the bone tumor. The resected brain tumor harbored T790M in addition to the exon 19 deletion. In contrast, T790M was not found in the specimen from the pulmonary metastatic tumor. These findings strongly suggest genetic instability and heterogeneity of the lung tumor in this case.

**Table 1:** The genetic heterogeneity of EGFR gene status in our patient.

Sampling Time	Origin	**Exon 19 deletion**^**a**^	L858R	T790M
At initial diagnosis	Bone	Positive	Negative	Negative

At initial diagnosis	Lung^b^	Negative	Negative	Negative

Three years after diagnosis	Brain	Positive	Negative	Positive

Five years after diagnosis	Lung^c^	Positive	Positive	Negative

Six years after diagnosis	Lung^c^	Positive	Negative	Positive

^a^I744-R748del, E749I (GAA to ATT), and A750K (GCA to AAA). ^b^Primary tumor. ^c^Pulmonary metastatic nodule.

We used oral TS-1 as the next regimen and achieved a good tumor response. However, she relapsed again. She received a salvage chemotherapy regimen comprising carboplatin and pemetrexed with bevacizumab, but this failed to inhibit tumor progression. We re-biopsied the pulmonary nodule by using computed tomography-guided needle biopsy. Gene analysis of the re-biopsy specimen revealed a unique deletion mutation in exon 19 and T790M. The L858R mutation was not found in this specimen. Subsequently, our patient received thoracic irradiation for the pulmonary nodule for locoregional control.

## Discussion

An EGFR double-activating mutation involving an exon 19 deletion and L858R was first reported by Sriuranpong *et al. *[8]. Subsequently, in a study involving 145 NSCLC patients, five cases of EGFR double-activating mutation were reported by Zhang *et al. *[9]. They also showed that the exon 19 deletion and L858R were located on the same allele. A double-activating mutation was also reported in Japan, found in four of 111 samples by Masago *et al. *[10]. Hata *et al. *analyzed complex mutations in the EGFR gene in 783 NSCLC patients [11]. They found 21 double mutations. The prevalence of the double-activating mutation was 0.3% to 3.6%. The biological role of the double-activating mutation with regards sensitivity to EGFR-TKI remains controversial.

The genetic background of tumor cells in lung cancer is not homogenous. Taniguchi *et al. *showed that NSCLC cells contain a heterogeneous population of both mutated and non-mutated EGFR tumor cells [12]. Jiang *et al. *also demonstrated the intratumor heterogeneity of EGFR mutations by using a microdissection-based method [13]. In addition, three independent studies also showed discordance in the EGFR gene status of the primary tumor and corresponding metastatic tumor. Kalikaki *et al. *showed that the EGFR mutation status differed between primary tumors and corresponding metastases in seven (28%) of 25 patients [4]. The other two studies reported identical results [5,6]. These findings clearly show the genetic heterogeneity of lung cancer.

There is increasing interest in how such genetic heterogeneity can be generated. The multicentric carcinogenesis hypothesis cannot fully explain this phenomenon. In the case of our patient, five independent EGFR clones, including the wild type, were identified. The four mutant clones had a unique consensus deletion mutation in exon 19 accompanied by two amino acid substitutions. If mutation occurs at random, the probability of an identical mutation occurring twice in the same patient would be nearly equal to the universal probability bound. Thus, all four tumor clones were from blood relatives with an identical genetic ancestor. In terms of the cancer stem cell hypothesis, cancers arise from stem cells that have accumulated oncogenic mutations for transformation [14]. The transformed stem cell transforms into the cancer stem cell, which is capable of self-renewal and differentiation. A tumor hierarchy consisting of a small number of quiescent cancer stem/progenitor cells and a large number of proliferating effector tumor cells may be generated. However, only a cancer stem cell can reproduce another cancer stem cell. Therefore, the genetic information found in tumors may be inherited from cancer stem cells. Sporadic and step-by-step accumulations of mutations in cancer stem cells can generate a variety of tumor clones with a distinct genetic background.

The sequential accumulation of *de novo *mutations in tumor cells makes it difficult to predict sensitivity to EGFR-TKI. It is possible that a tumor with wild-type EGFR before front-line chemotherapy can become an alternative tumor, harboring the EGFR-activating mutation. An accumulation of alternative mutations, including a T790M-insensitive mutation, in addition to the primary activating mutation could modulate the sensitivity to EGFR-TKI. Therefore, if available, frequent re-analyses of the EGFR gene status may be required during a long-term clinical course. Repeated biopsies often inflict pain on patients, especially those with NSCLC. Thus, physicians must carefully determine the need for additional sampling based on the risk and benefit to the patient.

## Conclusion

Spatial and temporal observation of the EGFR gene status could help in making an appropriate therapeutic decision for locoregional and systemic management of lung cancer. An alternative paradigm to determine a therapeutic strategy for a patient with lung cancer warrants consideration based on the genetic heterogeneity and instability of the tumor cells.

## Abbreviations

EGFR: epidermal growth factor receptor; EGFR-TKI: epidermal growth factor receptor tyrosine kinase inhibitor; NSCLC: non-small cell lung cancer; PNA-LNA PCR: peptide nucleic acid-locked nucleic acid polymerase chain reaction.

## Consent

Written informed consent was obtained from the patient for publication of this case report and any accompanying images. A copy of the written consent is available for review by the Editor-in-Chief of this journal.

## Competing interests

The authors declare that they have no competing interests.

## Authors' contributions

MO prepared the molecular genetic analyses and drafted the manuscript. TY and SN participated in drafting the manuscript. TS carried out the patient's therapy and helped to draft the manuscript. All authors read and approved the final manuscript.
